# *Carduus edelbergii* Rech. f. Mediated Fabrication of Gold Nanoparticles; Characterization and Evaluation of Antimicrobial, Antioxidant and Antidiabetic Potency of the Synthesized AuNPs

**DOI:** 10.3390/molecules27196669

**Published:** 2022-10-07

**Authors:** Shahid Jamil, Ghulam Dastagir, Ahmed Ibrahim Foudah, Mohammed Hamed Alqarni, Hasan Soliman Yusufoglu, Huda Mohammed Alkreathy, Ömer Ertürk, Muhammad Abdur Rehman Shah, Rahmat Ali Khan

**Affiliations:** 1 Department of Botany, University of Peshawar, Peshawar 25000, Khyber Pakhtunkhwa, Pakistan; 2 Department of Pharmacognosy, College of Pharmacy, Prince Sattam Bin Abdulaziz University, Al-Kharj 11942, Saudi Arabia; 3 Department of Pharmacognosy & Pharmaceutical Chemistry, College of Dentistry & Pharmacy, Buraydah Private Colleges, Buraydah 81418, Saudi Arabia; 4 Department of Pharmacology, Faculty of Medicine, King Abdulaziz University, Jeddah 21589, Saudi Arabia; 5 Faculty of Arts and Sciences, Department of Molecular Biology and Genetics, Ordu University, Ordu 52200, Turkey; 6 Department of Biotechnology, University of Science and Technology Bannu, Bannu 28100, Khyber Pakhtunkhwa, Pakistan

**Keywords:** antioxidant, antidiabetic, gold nanoparticles, antimicrobial, in vivo, in vitro, *Carduus edelbergii*

## Abstract

Background: Due to the high expense, less effectiveness and more side effects of available synthetic medicine, the researchers and communities are focusing on phyto-based natural bioactive compounds, which are considered safer for the treatment of syndromes and chronic diseases. Aim: The current project was aimed to determine the phytochemicals constituents available in the aerial parts of methanol extract of *Carduus edelbergii* via GC-MS, fabrication of AuNPs mediated with the mentioned extract; characterization and evaluation of antimicrobial, antioxidant and antidiabetic potency of the synthesized AuNPs. Methods: Confirmation of green synthesis of AuNPs, functional groups responsible for the reduction in Au^+^, size and crystallinity, morphology and quantity of gold (Au) were carried out by Ultraviolet-Visible (UV-Vis) spectroscopy, Transform Infrared (FTIR) spectroscopy, Scanning Electron Microscopy (SEM), X-ray Diffraction (XRD) and dispersive X-ray (EDX), respectively, whereas in vitro antioxidant characteristics were assessed by DPPH and ABTS assays. Wistar albino rats were used to test the anti-diabetic properties of the methanol extract and AuNPs. Results: GC-MS revealed that the diluted methanol extract of *Carduus edelbergii* consists of about 19 chemical constituents. Among the identified compounds, the 13-Docosenoic acid, methyl ester, (Z)—has the highest concentration (38.16%), followed by 9-Octadecenoic acid, methyl ester, (E)—(15.72%) and *n*-Hexadecanoic acid (15.07%). Methanol extract and its fabricated nanoparticles showed significant antioxidant and antimicrobial activities. In vivo antidiabetic study revealed a noteworthy (*p* < 0.05) decline in body weight and HDL and elevated concentration of blood glucose, bilirubin, creatinine, urea, triglyceride, VLDL, LDL, ALP, ALT and AST in diabetic control. The said changes were recovered significantly (*p* < 0.05) by treatment of diabetic rats with methanol extract (150 and 300 mg/Kg BW) and AuNPs of *Carduus edelbergii* (5 and 10 mg/Kg BW). Conclusion: The green synthesized AuNPs exhibit significant antioxidant, antimicrobial and antidiabetic characteristics.

## 1. Introduction

Although scientific advancement has been achieved in almost every field, the most promising area by far is nanotechnology in the 21st century, which has revolutionized almost every discipline of life.

Nanotechnology refers to the manipulation of size, shape and structure at the nanoscale in order to develop novel properties in materials in comparison to their bulk counterparts [1]. It can be broadly defined as the manipulation, production and designing of devices and structures at the nanoscale by controlling their shape and size in order to bring forth novel properties in them. The core building blocks of nanotechnology are nanoparticles (NPs), particles that range in size from 1 to 100 nm. The extremely small size and larger surface area enable NPs to exhibit properties that are not present in their bulk counterparts. Because of their physical, chemical, optical, magnetic, electrical, catalytic and thermal characteristics, metallic nanoparticles have biomedical, agricultural and environmental applications. Antibacterial metallic nanoparticles offer a possible alternative to conventional antibiotics [2].

There are many methods of stabilizing NPs, but they all fall into three basic groups: physical, chemical and biological methods. Physical techniques involve the practice of different techniques, including evaporation, condensation and laser ablation. Physical methods used for the synthesis of NPs are completely free of organic contaminants [3], but they require a lot of energy, a large space for the machinery involved, release a lot of heat and take a long time [3]. Reducing agents such as polyols, sodium citrate, ascorbic acid and Tollen’s reagents, elemental hydrogen, sodium borohydride and DMF (dimethylformamide) [4] are employed in chemical techniques to synthesize NPs. There are also certain drawbacks of the chemical method, which include the use of noxious, non-biodegradable chemicals. These chemicals may contaminate NPs’ surfaces making the NPs useless for the majority of biomedical applications. The said chemicals also have a damaging impact on the environment [5]. Biochemical or green synthesis of NPs is environmentally friendly and an alternative to physical or chemical techniques. It is less expensive, which makes it the method of choice. It can be executed at normal pH and room temperature, thereby reducing the time and efforts requisite to optimize temperature and pH for NPs synthesis. Biomolecules such as proteins and polypeptides, are derived from a wide variety of organisms have proved to be useful for NPs synthesis.

Nanomedicine is a sub-discipline of medicine that uses the fundamentals of nanotechnology to avoid and/or treat a number of sicknesses. In a living organism, nanomedicine entails the use of nanostructured materials for diagnosis, delivery, detection, or actuation [6]. Nanotechnology has the potential to improve medicine delivery to regions where macromolecules could not reach. Diabetes research is increasingly relying on nanotechnology. It is possible that it will provide information that is more accurate for the diagnosis of diabetes mellitus [7]. For the treatment of diabetes mellitus (DM) and its consequences, nanotechnology has recently emerged as one of the most important research fields. Within a range of 1–100 nm, the advantages of nano-materials include their versatility, control over size, and capacity to maintain their physiochemical properties. More surface functionalized active components can be encapsulated in nanomaterials with a higher surface area-to-volume ratio, which may facilitate cell adhesion and expedite particular regeneration processes [8]. Nanospheres as biodegradable polymeric carriers have been used in the oral delivery of insulin, the formation of artificial beta cells, and the creation of artificial pancreas [9].

Within the Asteraceae, the Cardueae (Thistle) is one of the largest tribes, primarily found in a temperate region. In Pakistan, this is found in Chitral, Swat, Kurram Valley, Gilgit, Astor, Baltistan, Kashmir and Northern Baluchistan. Carduus is known for its therapeutic virtues and is found primarily in Europe. The raw or cooked material of some Carduus taxa is used in traditional and modern medicines to treat liver illnesses, diuretics, digestives and rheumatism. Tonic, antispasmodic, anticancer, antibacterial and anti-inflammatory is just some of the functions of the secondary metabolites found in different species of this genus. Some of these plants are employed in Bulgarian folk medicine as diuretic, cardiotonic and antihemorroidal remedies [10].

Due to the astounding potential of green synthesized NPs in a number of scientific disciplines, contemporary research was intended to carry out the green synthesis of AuNPs using methanol extract of aerial parts of the plant, *Carduus edelbergii*. AuNPs are an important tool used in nanotechnology for a variety of purposes. AuNPs have found their applications in different areas of research, including catalysis, biosensors, bioimaging, delivery of drugs and photonics [11]. The biocompatibility of AuNPs is their most remarkable aspect, which render them an NP of preference for biomedical applications [12]. Different in vitro and in vivo tests are used to investigate the biological potentials of AuNPs fabricated with extract in this publication.

## 2. Results

### 2.1. GC-MS Analysis

**Figure 1:**
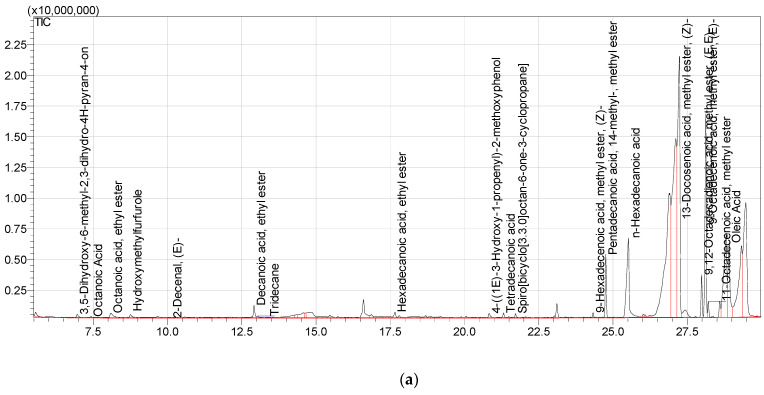

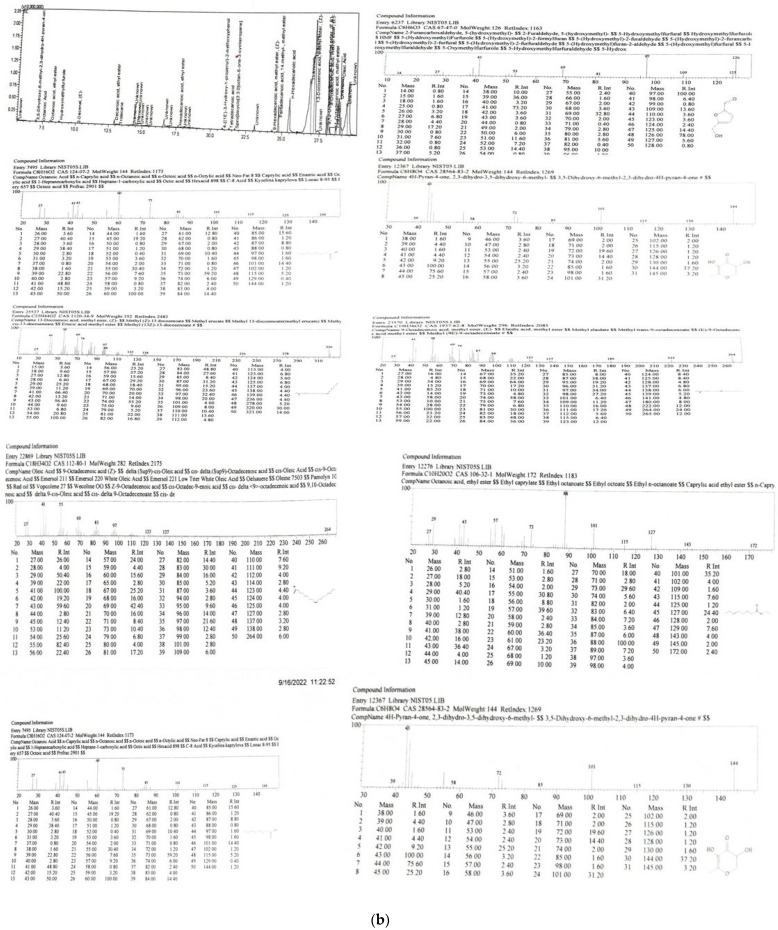
(**a**) Chemical constituents are found in the methanol extract of *Carduus edelbergii*, (**b**) total-ion chromatogram (TIC) calculated from GC-MS analysis of methanol extract of *Carduus edelbergii*.

**Figure 2:**
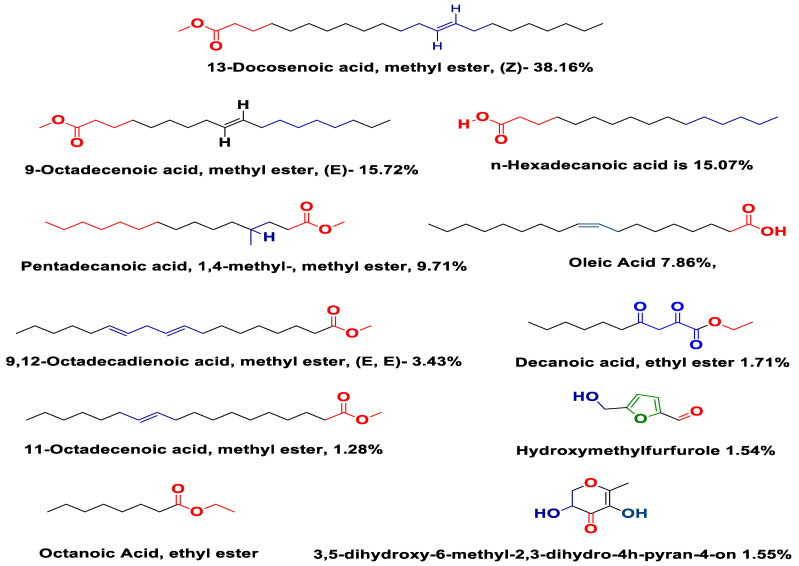
Chemical structure of chemical constituents showing higher concentration.

The GC/MS analysis of diluted methanol extract of aerial parts of Carduus edelbergii revealed that about 19 chemical constituents are found in the methanol extract of Carduus edelbergii (Figure 1a). The total-ion current (TIC) calculated from GC-MS is shown in Figure 1b. From the result, it was found that the esters were dominant, constituting 60% of the total mass, while other acids covered the remaining portion. Among the esters, 13-Docosenoic acid, methyl ester, (Z)—(fatty acid methyl ester) has the highest concentration (38.16%), followed by 9-Octadecenoic acid, methyl ester, (E)—(15.72%) in the mass of methanol extract. On the other hand, n-Hexadecanoic acid is 15.07% bymass. The Pentadecanoic acid, 1,4-methyl-, methyl ester; Oleic Acid; and 9,12-Octadecadienoic acid, methyl ester, (E, E)—constitute 9.71%, 7.86%, and 3.43%, of mass, respectively. Moreover, the Decanoic acid, ethyl ester (1.72%); 11-Octadecenoic acid, methyl ester (1.28%); 3,5-Dihydroxy-6-methyl-2,3-dihudro-4H-pyran-4-on (1.55%); Octanoic Acid, ethyl ester (1.05%); and Hydroxymethylfurfurale (1.54%) were found in the sample (Figure 2). The complete list of compounds names, percentage mass and calculated Kovit index is shown in Table 1.

### 2.2. Green Synthesis of AuNPs

A wide range of secondary metabolites is synthesized in plants and serve a variety of purposes. They have proved to be stabilizing and capping agents for nanoparticles, extending their shelf life and stability. The secondary metabolites from plant extract reduce the precursor metal salts into their respective metal ions. The metal ions are surrounded by phytochemicals that prevent the ions from recombining. The prolonged shelf life is also due to the phytochemicals surrounding the NPs, as they prevent the NPs from agglomeration. Thus, the synthesized AuNPs are more stable and effective than HAuCl_4_ and methanol extract. Secondary metabolites from the Carduus species are also known to be diverse. In the present study, the reaction of *Carduus edelbergii* extract to precursor salt solutions (HAuCl_4_.3H_2_O) changed the color of the resulting mixture indicating the instigation of NPs synthesis. Following the completion of process of NPs synthesis, they were separated and kept in Eppendorf tubes for further analysis.

#### 2.2.1. UV-Vis-Spectrophotometric Analysis

**Figure 3:**
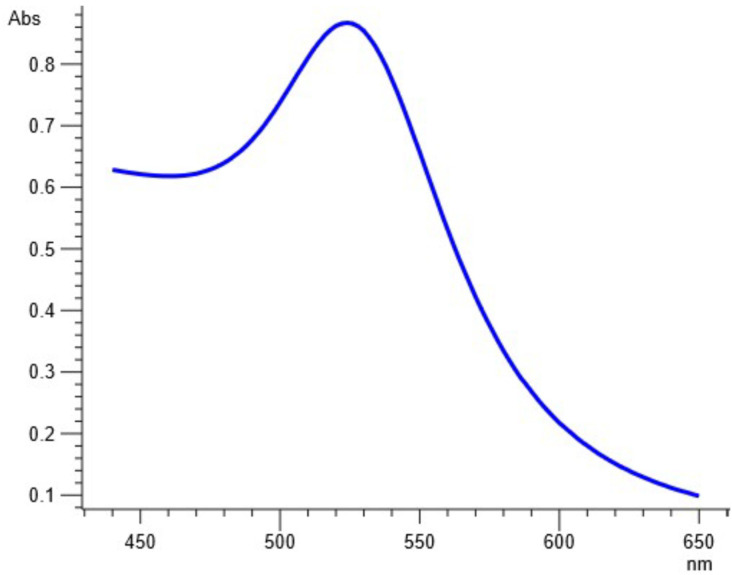
UV-Vis-Spectrophotometric Analysis of the *Carduus edelbergii* mediated AuNPs.

When utilizing the absorption spectrum of a sample, UV-vis spectrophotometry is a potent method that may be used to assess whether or not a sample contains metallic NPs. Spectrophotometric analysis of the produced AuNPs showed characteristic peaks (λ_max_) at 524 nm, as shown in Figure 3. Regarding AuNPs, the literature reveals that excitation of the surface plasmon vibrations gives a distinct peak in the range of 500–560 nm; however, the exact λ_max_ value usually depends upon the size of the formed AuNPs. Our result confirms the synthesis of AuNPs as a result of capping and stabilization by *Carduus edelbergii* extract.

#### 2.2.2. X-ray Diffraction Analysis

**Figure 4:**
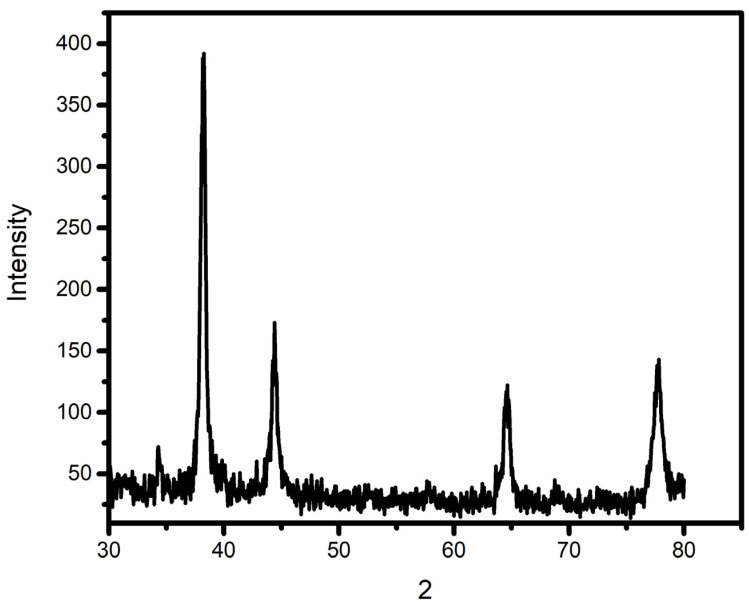
X-ray Diffractogram of the *Carduus edelbergii* mediated AuNPs.

The crystalline characteristics of AuNPs were determined with help of X-ray diffraction after the confirmation of their synthesis using UV-vis-spectrophotometry. Figure 4 reveals the XRD pattern of AuNPs. Distinct peaks were observed at 38.28°, 44.43°, 64.66° and 77.79°. These peaks correspond to 111, 200, 220 and 311 planes of face-centered cubic (fcc) crystal structure. The size of AuNPs was calculated to be 15.6 nm using the Debye–Scherrer equation.

#### 2.2.3. FR-IR Spectral Analysis

**Figure 5:**
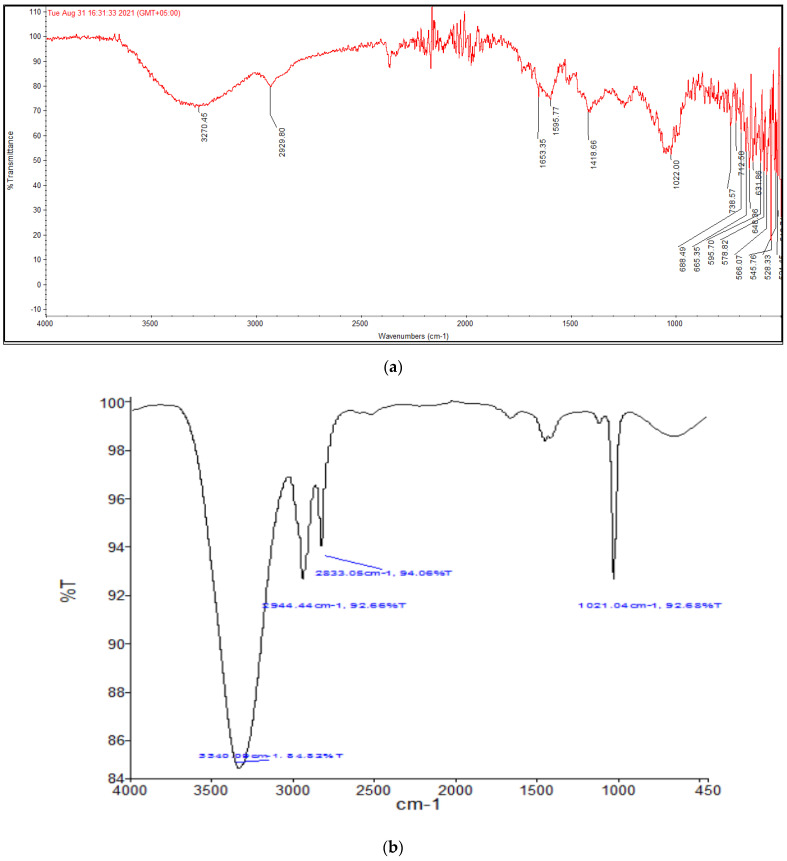
(**a**) FTIR of the *Carduus edelbergii* methanol extract, (**b**) FTIR of the *Carduus edelbergii* fabricated AuNPs.

The functional groups of the *Carduus edelbergii* plant extract were determined and predicted using FTIR measurements. The FTIR spectra of both plant extract and AuNPs were recorded (Figure 5a,b). FTIR analysis revealed the existence of several functional groups in the *Carduus edelbergii* extract. The broad peak at 3270.45 cm^−1^ can be attributed to the –OH functional group in alcohols and phenols. Similarly, the peak at 2929 cm^−1^ is attributed to the aliphatic sp^3^ C-H group. The peak at 1730 and 1653 cm^−1^ are due to the carbonyl and amide group, respectively, while the peak at 1408 cm^−1^ was assigned to –CN– stretching amines, and the peak at 1022 cm^−1^ was assigned to the C–N stretching vibration of the amine group.

The AuNPs synthesized showed relatively few distinct peaks. FTIR spectral analysis of the AuNPs showed peaks at 3340.09, 2944.44, 2833.05 and 1021.04 cm^−1^. This shows that out of the several phytochemical groups detected in the *Carduus edelbergii* extract, only limited phytochemicals were involved in the capping and stabilization of the AuNPs. The peak at 3340.09 can be attributed to the binding of phytochemicals containing the –OH group to the AuNPs, whereas the peaks at 2944.44, 2833.05 and 1021.04 cm^−1^ represent the C-H group and C-N stretching vibrations of amine group as already seen in the plant extract. The IR spectra of the synthesized AuNPs reveal altered and diminished peaks, which clearly imply that biomolecules of plants interacted with AuNPs, most probably via their oxygen functions, serve as capping and reducing agents.

#### 2.2.4. SEM Analysis

**Figure 6:**
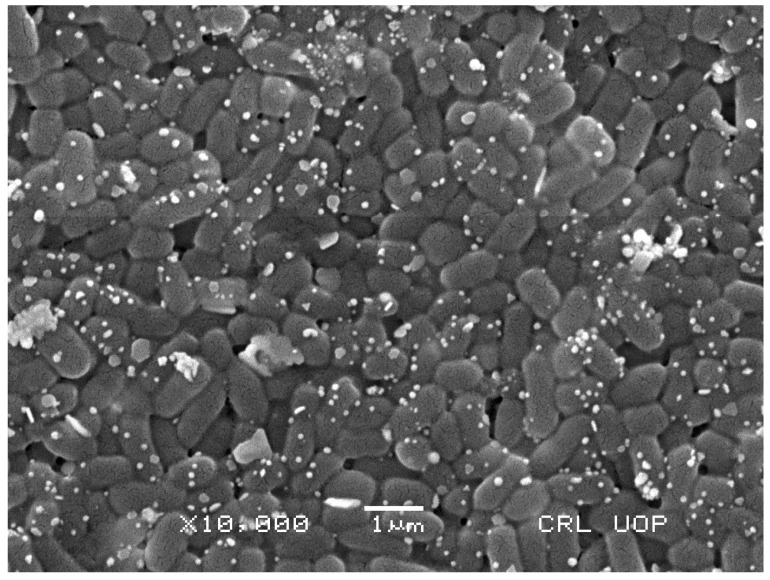
SEM analysis of the *Carduus edelbergii* mediated AuNPs.

SEM is a great tool used to examine the shape of AuNPs plant extract mediated nanoparticles. Figure 6 shows the SEM study of the AuNPs. It was revealed that the prepared particles are spherically shaped with the proper distribution. SEM micrograph revealed a certain degree of agglomeration in the synthesized AuNPs, which is common in NPs prepared using a biological route. The phytochemicals present in plant extracts are the primary reason behind a certain degree of agglomeration as compared to that of chemically synthesized NPs.

#### 2.2.5. EDX Study of Plant Mediated NPs

**Figure 7:**
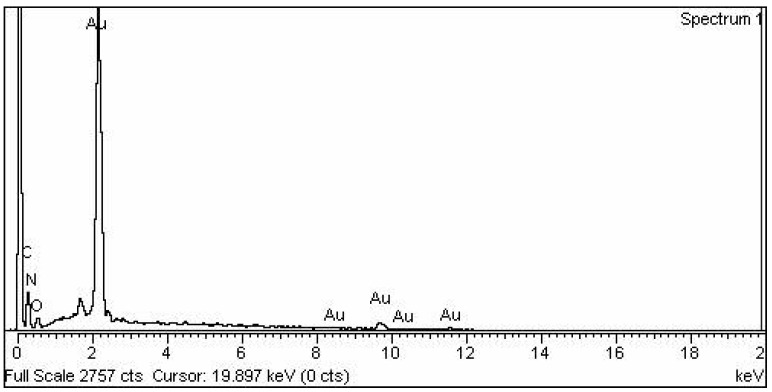
EDX elemental analyses of AuNPs.

The EDX analysis of the synthesized AuNPs is depicted in Figure 7. The elemental composition of the AuNPs sample is given in Table 2. In the case of AuNPs, EDX elemental analysis revealed a strong peak at 2 Kev of elemental gold, whereas certain other small peaks were observed between 8 and 12 Kev, which indicated the presence of gold in the sample (78.36% by weight). Other impurities, such as carbon and nitrogen, may be due to the application of plant extract as a capping and reducing mediator.

### 2.3. In Vitro Biological Investigations

#### 2.3.1. Antioxidant Potential

The antioxidant capability of *Carduus edelbergii* extract and its fabricated AuNPs were calculated through the scavenging potential of the extract and synthesized NPs against DPPH and ABTS free radicals. The findings are given below.

##### DPPH Assay

**Figure 8:**
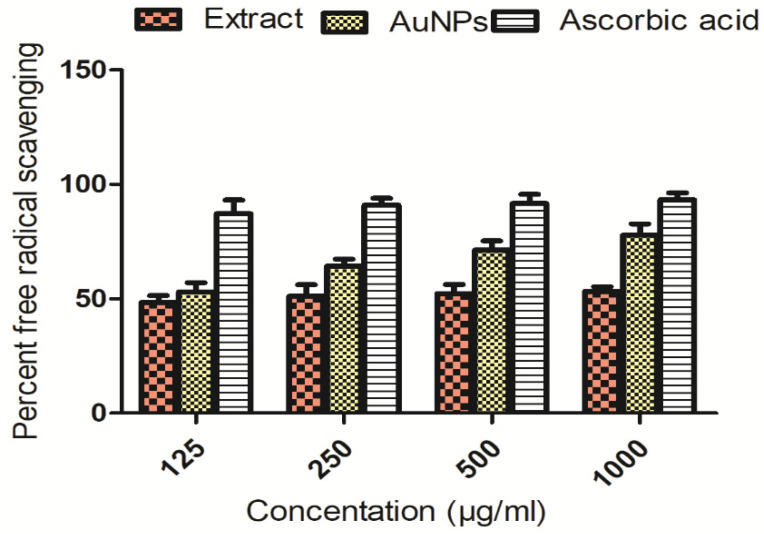
DPPH free radical scavenging effectiveness of extract and its fabricated AuNPs.

DPPH (2,2-diphenyl-1-picrylhydrazyl) free radicals were tested against extract-mediated AuNPs and plant extract, and the findings are shown in Figure 8. Maximum free radicals inhibitions were shown by extract, AuNPs and ascorbic acid at high concentration, i.e., 1000 µg/mL. The data obtained from triplicate experiments are presented as mean ± standard deviation.

##### ABTS Radical Scavenging Activity

**Figure 9:**
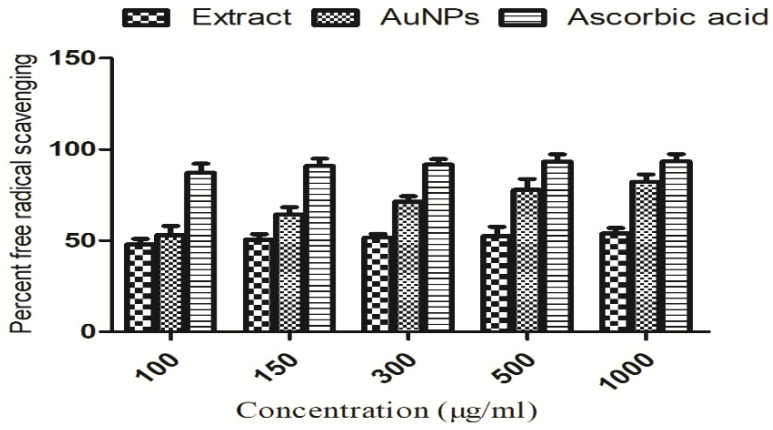
ABTS free radical scavenging capability of extract and its fabricated AuNPs.

AuNPs expressed better efficacy against ABTS (2,2′-azino-bis(3-ethylbenzothiazoline-6-sulfonic acid)) radicals (Figure 9) in a dose-dependent manner. The experiments were conducted in triplicate and expressed the data as mean ± standard deviation.

#### 2.3.2. Antimicrobial Activities

##### Antibacterial Activity

Green synthesized AuNPs have antibacterial activity against a variety of bacterial species. As shown in Table 3, AuNPs generated by *Carduus edelbergii* were effective in inhibiting the growth of bacteria such as *Klebsiella pneumonoae*, *Staphylococcus aureus, Escherichia coli*, *Staphylococcus epidermis* and *Acetobacter spp*. AuNPs established to be most effective against *K. pneumoneae* and *S. aureus* by showing a 12.3 ± 0.3 and 12.5 ± 0.6 mm zone of inhibition against both the aforementioned bacterial strains, respectively. Such astounding antimicrobial potential of AuNPs can be owing to their minuscule size, which accomplish their entry into the microbial cells easier. Upon entry, the NPs induce oxidative stress, which results in the destruction of microbial cells. NPs have a broad range of targets, i.e., induction of oxidative stress, disruption of the microbial cell wall, inhibition of vital enzymes and DNA damage, and hence NPs can efficiently inhibit the growth of microbes. AuNPs indicated admirable antibacterial effectiveness; 9.3 ± 0.4, 10.7 ± 0.6 and 11.2 ± 0.6 mm against *S. epidermis*, *E. coli* and *Acetobacter spp.* respectively. The results are comparable to that of the standard (Amoxicillin), for which the zones of inhibition recorded were 16.2 ± 0.5 for *S. epidermis*; 14.1 ± 0.7 for *S. aureus*; and 15.3 ± 0.4, 15.2 ± 0.3 and 15.4 ± 0.2 mm each for *K. pneumoneae*, *Acetobacter spp.* and *E. coli*, respectively.

##### Antifungal Activity

The development of fungi in test tubes can be used to determine the NPs’ fungicidal potency. All strains were obtained from UST Bannu’s Dept. of Biotechnology for antifungal assessment. The current research was based on the utilization of two strains of fungi; *Aspergillus niger* and *Aspergillus flavus*. The percent inhibition of the examined fungi served as the basis for calculating the antifungal activity (Table 4). *Aspergillus flavus* and *Aspergillus niger* were inhibited by AuNPs at 92.4 ± 4.3 and 70.8 ± 5.5 percent, respectively, compared to terbinafine 97.5 ± 2.5 and 98.1 ± 1.9 percent inhibition for both fungal strains. The antifungal potency of plant extracts is likewise impressive, with 62.28 ± 2.7 percent for *Aspergillus flavus* and 58.3 ± 2.7 percent for *Aspergillus niger*.

##### Minimum Inhibitor Concentration of Plant Extract and AuNPs against Bacteria

The extract fabricated AuNPs revealed the lowest MIC (2.50 µg/mL) against *Staphylococcus aureus* and the highest (25.13 µg/mL) against *Klebsiella pneumoneae,* as shown in Table 5. Overall, the MIC of plant extract is higher than AuNPs (Table 5).

##### Minimum Inhibitory Concentration of Plant Extract and AuNPs against Fungi

The minimum concentration of AuNPs that inhibit *Aspergillus niger* was determined as 6.31 µg/mL. Similarly, the MIC of plant extract against *Aspergillus flavus* (10.75 µg/mL) is lower than *Aspergillus niger* (13.50 µg/mL), as indicated in Table 6.

#### 2.3.3. Alpha-Amylase Inhibition

The findings of alpha-amylase assay are indicated in Table 7. The mentioned characteristics of existing marketable medicine, Glucophage (standard), methanol extract and AuNPs fabricated with extract were measured at 60.8 ± 3.8%, 65.1 ± 3.2% and 63.7 ± 5.1%, respectively. The experiments were conducted in triplicate (*n* = 3).

### 2.4. In Vivo Study

#### 2.4.1. Test for Acute Toxicity

No acute toxicity symptoms were observed in rats after a single oral dose of *Carduus edelbergii* methanol extract (2 g/Kg BW). Up to 2 g/Kg BW *Carduus edelbergii* extract is considered safe, with an estimated LD_50_ of greater than 2 g/Kg BW.

#### 2.4.2. Effect of a Methanol Extract and AuNPs on Body Weight of Diabetic Rats

On the first and 21st days of treatment, there was a substantial (*p* < 0.05) weight loss in the diabetes control group compared to the normal control group. In comparison to diabetic controls, treatment of diabetic rats with a methanol extract of *Carduus edelbergii* reliably (*p* < 0.05) restored their weight (Table 8). After 21 days of treatment, rats with diabetes showed a significant rise in body weight as compared to the untreated diabetic rats after taking the extract at 300 mg/Kg BW as well as AuNPs (10 mg/Kg BW).

#### 2.4.3. Effect of Methanol Extract and AuNPs on Blood Glucose

Treatment of diabetic rats with aerial parts of *Carduus edelbergii* extract and AuNPs revealed a considerable (*p* < 0.01) decline in the concentration of their blood glucose (Table 9); hence, the applied samples were presented as hypoglycemic agents. Conversely, the diabetic control group had a higher level of blood glucose during the experiment. The rats treated with a higher dose (300 mg/Kg BW) of extract, as well as AuNPs (10 mg/Kg BW) shown in Table 9, have an observable decline in the concentration of glucose in blood.

#### 2.4.4. Effect of Methanol Extract and AuNPs on Rat’s Liver Function

Alloxan has been shown to harm the pancreas and other organs, such as the liver and kidneys, in laboratory rats. Liver damages were assessed by measuring the levels of biochemical markers in the blood: AST, ALT, total bilirubin and ALP, all of which are highly sensitive indicators of oxidative stress and toxic substances. Table 10 shows the protective effects of glibenclamide, a methanol extract of *Carduus edelbergii* and AuNPs against alterations in liver serum markers. *Carduus edelbergii* extract significantly lowered the activity of marker enzymes in liver serum in rats treated with alloxan compared to the control group (*p* < 0.05). Treatment with 10 mg/Kg BW glibenclamide, as well as AuNPs, reduced alloxan intoxication considerably (*p* < 0.05).

#### 2.4.5. Effect of Methanol Extract and AuNPs on Total Cholesterol, Triglycerides, LDL Cholesterol, Serum VLDL Cholesterol and HDL Cholesterol 

In the presence of hepatotoxins and polyunsaturated fatty acids, free radicals lead to lipid peroxidation and alter the lipid profile. Triglycerides, very low-density lipoprotein (VLDL), low-density lipoprotein (LDL) and total cholesterol were all higher in the serum of diabetic rats, whereas HDL cholesterol was lower (Table 11). *Carduus edelbergii* extract (150 and 300 mg/Kg BW), as well as AuNPs, reliably recovered these values depending on the dose. The optimum dose provides the maximum therapeutic effects. Increasing the concentration of the drug up to the optimum level encounters a greater number of target cells, molecules or antigens, and hence better health recovery.

#### 2.4.6. Effect of a Methanol Extract and AuNPs on Creatinine and Urea in Rat’s Serum

Table 12 illustrates the alterations in serum total protein, creatinine and urea concentrations in normal, diabetic and treated groups. At 150 and 300 mg/Kg BW, *Carduus edelbergii* extract, as well as AuNPs, the elevated serum level of the renal profile was significantly (*p* < 0.05) restored; urea, serum creatinine and a decline in total serum protein in the treatment groups were noted.

## 3. Discussion

The phytochemical analysis showed that the extract of the aerial parts of *Carduus edelbergii* Rech. f. contains a mixture of bioactive compounds that were reported to have therapeutic effects [13]. It was shown in the result that the methanol extract of *Carduus edelbergii* is full of useful constituents. Among the esters, 13-Docosenoic acid, methyl ester, (Z)—(fatty acid methyl ester) has the highest concentration (38.16%), followed by 9-Octadecenoic acid, methyl ester, (E)—(15.72%) in the mass of methanol extract. On the other hand, n-Hexadecanoic acid is 15.07% in mass. The Pentadecanoic acid, 1,4-methyl-, methyl ester; Oleic Acid; and 9,12-Octadecadienoic acid, methyl ester, (E, E)—constitute 9.71%, 7.86% and 3.43% of mass, respectively. Moreover, the Decanoic acid, ethyl ester (1.72%); 11-Octadecenoic acid, methyl ester (1.28%); 3,5-Dihydroxy-6-methyl-2,3-dihudro-4H-pyran-4-on (1.55%); Octanoic Acid, ethyl ester (1.05%); and Hydroxymethylfurfurale (1.54%) were found in the sample. Congruent results were shown by *Carduus pycnocephalus* which exhibited hexadecanoic acid (39.62%), Pentadecanoic acid methyl ester (0.43%), 9-octadecenoic acid methyl ester (1.44%) [14].

Ali, R. et al., reported 13-docosenoic acid with a concentration of (34.06%) in *Acacia nilotica* [15]. All the above-given references mentioned the biological importance of identified compounds.

As soon as NPs are synthesized, it is critical to describe them to obtain a good picture of their physical and chemical properties. Since various factors such as the shape, size, crystallinity and morphological features of NPs influences their biological potential, it is important to have an understanding of these features and their effect on their biological potential. Various characterization techniques such as UV-vis spectrophotometry, XRD, FTIR, SEM and EDX were utilized to characterize extract-mediated AuNPs. UV-vis spectrophotometric analysis confirmed the presence of AuNPs in the crude sample, which was then isolated through centrifugation for further characterization. XRD analysis was used to determine the crystallinity and particle size. These peaks obtained in the XRD image correspond to 111, 200, 220 and 311 planes of face-centered cubic (fcc) crystal structure. The results proved that extract-mediated AuNPs were crystalline in nature, with a crystal size of approximately 15.6 nm. The XRD results of AuNPs are in concurrence with earlier studies on *Carduus crispus*. From the FTIR spectral analysis, it is evident that the functional groups detected in *Carduus edelbergii* extract were accountable for the capping and stabilization of AuNPs since the peaks in AuNPs samples are almost at the same position as in the plant extract sample. A small change in the position of the peaks and the intensity of the peaks may be attributed to the reaction of plant phytochemicals with the precursor salt to produce AuNPs. Relatively few peaks in AuNPs sample as compared to plant extract means that most of the phytochemicals exist in the plant extract remained unbound and did not play a part in the capping of AuNPs. The results of our study are similar to other studies [16].

SEM and EDX analysis also gave useful insights regarding the morphology and purity of the extract-mediated AuNPs. The AuNPs obtained were morphologically spherical. A high degree of agglomeration can be seen in the XRD image, which is quite understandable considering the involvement of various phytochemicals in the capping of synthesized AuNPs. EDX elemental analysis revealed a strong peak of elemental gold, which indicates the presence of gold in the sample. The use of plant extract as a capping and reducing agent may have caused other impurities such as carbon and nitrogen.

The higher potential for improving health care that nanosized molecules have than those already found in nature could make the discovery and manipulation of new desirable molecules [17]. In the last few years, several pharmaceutical companies have received FDA approval for the usage and development of medications based on nanotechnology. The capacity of gold compounds to decrease NF-kappa B expression and associated inflammatory reactions has garnered considerable interest as an anti-inflammatory drug [18]. Animal studies in cerebrally ischemic rats have shown that Swarna Bhasma contains immunomodulatory, antioxidant, and restorative properties that could be useful in treating ischemia and other damage to the central nervous system [19]. Because ionic gold is easily complexed and precipitated, it has a limited ability to perform its intended tasks in the human body. Zerovalent gold nanoparticles can be an excellent substitute for metallic gold in these situations. One-step green production of nanoparticles from metal ions can be achieved using biomolecules found in plant extracts. At room temperature and pressure, the biogenic reduction in a metal ion to a base metal can be easily scaled up. Thus, the green-produced gold nanoparticles have both zerovalent gold and a bioactive component, giving superior curative effects for the treatment of cancer. Owing to its biocompatibility and strong surface reactivity, gold nanoparticles (AuNPs), a developing nanomedicine, are known for their promising therapeutic capabilities [20]. VPF/VEGF165-induced endothelial cell proliferation is inhibited by gold nanoparticles, providing compelling evidence that they can be used as therapeutic agents for a wide range of conditions, including rheumatoid arthritis and cancer. In light of the evidence that traditional gold has an antioxidative effect in the treatment of various inflammatory diseases and other related disorders that are context-dependent, the need for further research into the restorative effect of gold nanoparticles in conditions of hyperglycemia has been reported in previous studies [21].

The antimicrobial potency of nanoparticles is very much successful and largely applied. In the current study, AuNPs proved to be most effective against *K. pneumoneae and S. aureus* by showing 12.3 ± 0.3 mm and 12.5 ± 0.6 zone of inhibition against both the aforementioned bacterial strains, respectively. AuNPs indicated brilliant antibacterial potency against *S. epidermis*, *E. coli* and *Acetobacter* spp. 9.3 ± 0.4, 10.7 ± 0.6 and 11.2 ± 0.6 mm each, respectively. *Aspergillus flavus* and *Aspergillus niger* were inhibited by AuNPs at 92.4 ± 4.3 and 70.8 ± 5.5 percent, respectively, compared to terbinafine 97.5 ± 2.5 and 98.1 ± 1.9 percent inhibition for both fungal strains. The antibacterial characteristics of AuNPs were also checked with their MICs and found that the sample with the lowest MIC had the highest inhibition zone. Congruent findings have been recorded in earlier studies [22].

Alteration in carbohydrate metabolism may cause serious health problems such as diabetes and obesity, where a decline in function or secretion of insulin leads to an increase in blood glucose level and hence the ultimate onset of diabetes mellitus [23]. The inhibition of alpha-amylase causes a delay in the digestion of carbohydrates and, thus, a decline in glucose uptake by the intestines. In order to determine the antidiabetic potential of plant extract, the in vitro alpha-amylase inhibitory assay was opted for. The anti-diabetic characteristics of AuNPs (63.7 ± 5.1) were higher than extract (60.8 ± 3.8) and were comparable with Glucophage (65.1 ± 3.2). This predicts that the plant extract is an effective source of antidiabetic phytochemicals. The synthesis of extract fabricated AuNPs showed improved antidiabetic activities. Analogous results were found during in vitro studies of *Carduus lanuginosus* [13] and *Carduus marianum* [24] extract. In addition, plants protect themselves with the help of their indigenous alpha-amylase inhibitors. The mentioned inhibitors alter the digestive capability of proteinases and alpha-amylases in the gut of insects and decrease their normal feeding activities [25]. The greater alpha-amylase inhibitory potential was revealed by AuNPs and methanol extract; they were subjected to in vivo investigation and obtained desirable results. The antioxidant, antihyperglycemic and hypolipidemic properties of phytochemical compounds were reported [26]. Enzymatic functions can also be restored by curing pancreatic islets and revamping organs such as the liver and kidneys. Diabetes-induced alterations in diabetic rats were largely reversed by *Carduus edelbergii* methanol extract. The *Silybum marianum* extract prevents diabetic rats from losing weight by restoring glucose levels, enzymes and tissue damage [24]. *Carduus acanthoides* anti-diabetic investigations provided similar results in the past [27]. Aminotransferase levels tend to rise in patients with diabetes and are more common than in the general population [28]. After the introduction of alloxan in prior research, there was a large rise in the activity of specific enzymes, such as Beta-Glucuronidase, Nacetyl-bet Glucosaminidase, Leucine Aminpeptidase, Lysosomal Acid Phosphates, and Cathepsin D [29]. Diabetes-related alterations in metabolism are closely linked to serum enzyme attenuation in animals. As a result of the increasing availability of amino acids in diabetes, transaminase activity rises, resulting in an increased rate of ketogenesis and gluconeogenesis. As a result, the better metabolism of carbohydrates, lipids and proteins led to a recovery in enzyme levels. After treatment, bilirubin and ALT levels improved, indicating that insulin secretion was restored. In previous studies, the plant extract showed restorative properties of ALT, ALP, total bilirubin and AST [30]. As a result of diabetes, a raise in serum creatinine and urea was seen, which were considered major indicators of renal impairment [31]. A result from anti-diabetic research of *Carduus acicularis* and *Cirsium maackii* was consistent with our results [32]. Rats suffering from alloxan-induced diabetes were shown to have hypercholesterolemia, which can be attributed to a drop in fat-catalytic hormones and a reduction in the breakdown of fat deposits [33]. There may be an association between the lipid-lowering impact and the suppression of the formation of fatty acids. Lipoprotein lipase, which hydrolyzes triglycerides, is activated by insulin during metabolism, but insulin shortage causes insulin to be inactivated, resulting in hypertriglyceridemia [34]. A considerable decline in serum lipid concentrations in diabetic rats subsequent to treatment with methanol extract can be directly linked with an enhancement in insulin concentrations. Comparable findings were acquired in an antidiabetic study of the aerial parts of *Iphiona aucheri* extracts [25].

## 4. Materials and Methods

### 4.1. Collection and Identification of Plant

Fresh samples of aerial parts of *Carddus edelbergii* were gathered from Bajaur, Khyber Pakhtunkhwa, Pakistan, identified by Prof. Dr. Ghlum Dastagir, Chairman Department of Botany and allotted Voucher number Shahid Jamil Bot 180 (PUP). Distilled water was used for washing collected plant materials to eliminate dust particles and other impurities and then shade dried for 25 days in the summer season, pulverized and protected in sealed flasks for further use.

### 4.2. Extract Preparation

The extract was prepared by adding methanol to powdered plant material in 3:1 (*v/w*) and shook well. After 7 days, the sample was subjected to a rotary evaporator to obtain a more concentrated crude extract which was kept at 4 °C until future applications.

### 4.3. GC-MS Analysis

GC-MS analysis of particle-free diluted 1 µL methanol extract was carried out on PerkinElmer Clarus 600 GC System in accordance with the established procedure followed by Naz et al., [35].

### 4.4. AuNPs Green Synthesis

As a precursor salt, HAuCl_4_·3H_2_O and plant extract were utilized to make AuNPs. In order to produce AuNPs, a solution of HAuCl_4_·3H_2_O (95 mL) was combined with plant extract dissolved in 5 mL methanol, and the mixture was left to incubate for twenty-four h at room temperature. Thereafter, the NPs were separated from the remainder of the material by using a centrifuge, Spectrafuge 24D (Labnet International, Inc., Edison, NJ, USA). The centrifugation was performed at 12,000 rpm for 12 min in 2 mL Eppendorf tubes. Removal of the supernatant resulted in the isolation of AuNPs from the mixture. In order to eliminate any unattached phytoconstituents and unreacted precursor salt solution, the AuNPs were extensively rinsed with ddH_2_O. The rinsing was followed again by centrifugation to remove any unbound materials. The rinsing procedure was repeated four times so that all the unbound substances were removed.

### 4.5. Characterization of NPs

#### 4.5.1. Spectrophotometric Analysis

The Halo DB-20 UV-Vis Double Beam Spectrophotometer was used in this work. To scan the reaction mixtures, we used a wavelength (max) range of 440–650 nm. The occurrence of a maximum between 500 and 560 indicates the presence of AuNPs.

#### 4.5.2. X-ray Diffraction (XRD)

An X-ray Diffractometer in the 10–80° temperature range with a scanning step size of 0.03°/s and a step time of 0.55 s was used to examine the crystallographic structure of AuNPs made using extract (Model-D8 Advance, Karlsruhe, Germany). For the purpose of gathering information, data were gathered using Cu K radiation (wavelength 1.5406 nm, generator voltage of 40 kV, tube current 30 mA). We used powdered NPs (1 mg) for the experiment. The size of NPs was calculated by using Debye–Sherrer formula as follows.

D = k λ/βCosθ(1)

where:

λ = X-rays wavelength (1.5421 Å);

θ = Bragg’s Angle;

k = Shape Factor (0.94);

β = Full width at half maximum in radians.

#### 4.5.3. Fourier Transformed Infrared Spectroscopy (FTIR)

The FTIR spectrometer (Spectrum One, Perkin Elmer, Waltham, MA, USA) was used to identify the functional groups attached to the produced NPs using KBr pellets at a spectrum range of 500 to 3500 cm^−1^.

#### 4.5.4. Scanning Electron Microscopy (SEM) Energy Dispersive X-ray (EDX) Analysis

SEM (Jeol JSM-6510LV) was used to examine the surface morphology of the produced NPs. Nanoparticles were attached to a support, and gold sputtering was used to enhance the ionic conductivity of the NPs. Elemental analysis was accomplished by the use of EDX technology. For this purpose, the sample was coated with carbon, dried, and detected with an EDX detector connected to SEM.

### 4.6. In Vitro Biological Activities

#### 4.6.1. Antioxidant Characteristics

The antioxidant potential of *Carduus edelbergii* was determined using the following assays.

##### DPPH Free Radical Scavenging Assay

The antioxidant effects of the prepared NPs were tested using the DPPH free radical scavenging assay according to Gyamfi et al. [36] protocol. The stock solution was prepared by dissolving 3 mg DPPH in 100 mL methanol and its absorbance was adjusted to less than one (0.980) at 517 nm. Samples of various concentrations (125 to 1000 µg/mL) were combined with 1800 µL DPPH solution. Following the incubation for 15 min, measured the absorbance at 517 nm and calculated the DPPH scavenging activity as follows.

Percentage inhibition = Absorbance of control − Absorbance of sample/Absorbance of control × 100(2)

##### ABTS Free Radical Scavenging Assay

ABTS experiment was performed in accordance with the reported protocols [34]. The stock solution was prepared by combining 7 mM ABTS and 2.4 mM potassium persulfate solutions. The resultant mixture was kept at 37 °C for 12–16 h in full darkness before being diluted with methanol (1:1) and tested for absorbance until it reached 0.70. The absorbance was measured with the help of spectrophotometer at 734 nm. According to the following formula, the percentage of inhibition was determined:

Percentage inhibition = [Absorbance of control − Absorbance of sample/Absorbance of control] × 100(3)

#### 4.6.2. In Vitro Investigation of Antidiabetic Activity through α-Amylase Inhibition

The Worthington Enzyme Manual [37] was practiced to examine the inhibitory characteristics of extract and AuNPs fabricated with plant extract against α-amylase. Pre-incubation was performed at 25 °C for 10 min using a mixture of extract (300 µL), starch (1 percent) and α-amylase solutions (0.5 mg/mL). After incubation in boiling water for five minutes and cooling to room temperature, the enzymatic process ceased by adding 1.0 mL of dinitrosalicylic acid (DNS), a color reagent. Sodium phosphate buffer was used to prepare the starch, α-amylase, and DNS acid solutions (20 mM, pH 6.9 with 6 mM NaCl). Each test tube was diluted with 3 mL of distilled water. The absorbance of blank (control) containing buffer as a substitute of plant extracts and experimental was measured at 540 nm spectrophotometrically. The activity of control was considered 100%. The % inhibition of α-amylase was measured using the following formula:

Amylase inhibition (%) = [(control absorbance (blank)/sample absorbance)/(control absorbance)] × 100(4)

#### 4.6.3. Antimicrobial Assay

##### Antibacterial Activity

The antibacterial activities of extract and AuNPs were evaluated against five distinct bacteria, including *Klebsiella aerogenes* (KPC), *E. coli*, *Staphylococcus epidermis* (SEP), *Staphylococcus aureus* (SA) and Acetobacter species (Ac) by using agar well diffusion assay. Cotton swabs were used to inoculate bacterial strains onto agar plates. Aliquots of 20 microliters were obtained from each sample, including extract (10 mg/mL), standard antibiotic (Amoxicillin), AuNPs and DMSO. Prior to the experiment, all of the equipment was sterilized thoroughly. Over the course of 24 h, plates were left to soak up the nutrients in an incubator at room temperature. Measurements were made in millimeters after 24 h of incubation.

##### Antifungal Activity

Slant Technique Antifungal Activity: Slant method antifungal activity was performed. An amount of 3.5 g of SDA was dissolved in 50 mL water in a conical flask with cotton to prepare SDA media. Autoclaving for 15 min at 121 °C and 15 psi pressure sanitized all of the media, test tubes and wire loops used in this experiment. The slants were prepared in a laminar flow hood by putting SDA (60 mL) into test tubes. Aliquots from each sample (i.e., extract-mediated AuNPs (10 mg/mL), standard antibiotic (Terbinafine; Saffron Pharmaceuticals Pvt. Ltd., Faisalabad, Pakistan.), and DMSO solution were poured into the relevant wells and incubated for 30 min at 37 °C.

##### Minimum Inhibitory Concentrations (MICs) Determination

The MICs of *Carduus edelbergii* extract and its fabricated AuNPs were determined by using agar well diffusion method, and their efficiencies were evaluated by inhibiting the growth of food pathogens and spoiling microorganisms [38]. Serial dilutions were used to create concentrations ranging from 20 to 1.25%. Agar was added to sterile Petri dishes, and 1 mL of each prepared inoculums was pipetted onto the agar. Then, on each plate, five wells were formed, each containing 100 μL of extracts containing 20, 10, 5, 2.5 and 1.25 mg/mL. At 37 °C, plates were kept in the refrigerator for 30 min before incubating for 18 h. Around 10–12 fungal spores were put on the slanted surface of SDA media in the test tubes to determine the minimum inhibitory concentration of each sample. Triplicates of each assay were carried out.

### 4.7. In Vivo Antidiabetic Evaluations

#### 4.7.1. Experimental Animals 

Male Wistar Albino rats (260–280 g) were purchased from the animal house at the National Institute of Health Sciences (NIH), Islamabad, for use in this investigation. All of the animals were housed in cages at a temperature of 25 ± 0.5 °C, with a 12 h light/dark cycle and fed a locally purchased pellet diet and fresh tap water, according to standards approved by the Institutional Animals Ethics Committee (IAEC number 131). For one week before the initiation of a trial, the rats were acclimatized in Bannu to the laboratory environment.

#### 4.7.2. Toxicity Study

Six healthy male Wistar Albino rats from each of the two test groups and the reference group were fasted overnight and given free access to water. The experimental groups were given different doses (1 and 2 g per kilogram of body weight) of methanol extract of the *Carduus edelbergii* in order to determine the plant’s potential toxicity. Experiment animals were kept alive for 24 h, and their behaviors were monitored for any signs of acute toxicity, but they were not found. It revealed that LD_50_ of tested extract >2000 mg/Kg BW. By keeping in view the mentioned test, specified doses (150 and 300 mg/Kg BW) of extract and (5 and 10 mg/Kg BW) of AuNPs were chosen for the evaluation of its anti-diabetic properties. 

#### 4.7.3. Induction of Diabetes in Rats

Each experimental rat was given a single intra-peritoneal injection of freshly prepared alloxan (120 mg/Kg BW) in a volume of 1 mL/Kg BW in normal saline to induce experimental diabetes [39]. Following a 72 h, the blood glucose levels of rats were tested, and those with a fasting blood glucose level ≥ 200 mg/dL was declared diabetic [34] and included in the study as planned subjects.

#### 4.7.4. Experimental Procedure

After the rats were proven to be diabetic, they were arbitrarily separated into five groups of five rats each. A permanent marker was used to number and label the tails of all the rats in a particular group. Diabetic rats were treated with glibenclamide at 10 mg/Kg BW. In group 3, rats were administered methanol extract of the *Carduus edelbergii* at 150 mg/Kg BW. The group 4 rats were administered with methanol extract at 300 mg/Kg BW. Group 5 rats were administered with glibenclamide at 10 mg/Kg BW. By using a 16 gauge stomach intubation, glibenclamide, plant extract and AuNPs were delivered orally to rats daily for 21 days to imitate the traditional oral use of ethnomedicinal remedies for a range of disorders.

#### 4.7.5. Measurement of Blood Glucose Concentration and Body Weight

Fasting blood glucose levels were measured with a glucometer prior to the administration of the first dose (t = 0; day 1) and on the first, seventh, fourteenth and twenty-first days of treatment (Roche USA). Blood samples from rats were taken by piercing their tail veins with an aseptic needle. Their first (t = 1st day) and finishing (t = 21st day) weights were measured and articulated the changes.

#### 4.7.6. Sacrificing Animals and Serum Collection

Following the 21st day of treatment with extract and its fabricated AuNPs, the rats were anesthetized and rendered unconscious by inhalation of the anesthetic agent and diethyl ether (anesthetic ether), and they were then slaughtered. The blood taken by piercing the heart was maintained in plain simple bottles, permitted for two h to clot and then for 10 min centrifuged at 3000 rpm to get serum. A sample of the serum (the supernatant) was taken for further study.

#### 4.7.7. Lipid Profile Determination of Serum

A chemical analyzer (Selectra, XL, Dieren, The Netherlands) and available kits (Gesan products, Campobello di Mazara, Italy) were used to determine the levels of total cholesterol, HDL and triglycerides (TG) in blood serum. The Friedewald equation was used to estimate VLDL and LDL cholesterol levels in the bloodstream.

#### 4.7.8. Judgment of Kidneys and Liver Function

A chemistry analyzer (Selectra, XL, Dieren, Netherlands) and available kits (Gesan productions, Campobello di Mazara, Italy) were used to determine aspartate and alpha-alanine aminotransferase (ALT and AST), creatinine and total bilirubin.

#### 4.7.9. Determination of Malondialdehyde (MDA)

A TBARS assay was performed in accordance with the standard methodology opted by Shah et al., to examine lipid peroxidation [34]. After 10 min of centrifugation at 4000× *g*, the supernatant from the 10 percent homogenate of liver tissue was recovered. An amount of 100 µL of supernatant was added in test tubes without extract (control) and samples (experimental) and maintained at 37 °C for 60 min. TBA solution, acetate buffer and 8.1 percent SDS were added to the mixture and incubated for 60 min at 100 °C. TBA’s response with MDA was evident by the emergence of a light pink color. After ice cooling the tubes, the absorbance was measured spectrophotometrically at 532 nm.

### 4.8. Statistical Analysis

The data were analyzed using GraphPad Prism. As a result of these studies, the mean standard deviation was calculated for all of the in vitro and in vivo investigations. Total phenolic content was compared to antioxidant and antidiabetic properties using the Pearson correlation coefficient. In vivo testing was performed using one-way ANOVA and Dunnett’s-tests. *p* < 0.05 was considered statistically significant.

## 5. Conclusions

The current research project suggests that the methanol extract of aerial parts of *Carduus edelbergii* Rech.f. and its fabricated AuNPs exhibit significant antimicrobial, antioxidant and anti-diabetic activities. The AuNPs were effectively synthesized from the bio-reduction in HAuCl_4_ using *Carduus edelbergii* Rech.f. methanol extract. The NPs were confirmed and characterized using different methods, viz., UV-Vis spectroscopy, FTIR, XRD, Scanning Electron Microscopy (SEM) and EDX analysis. The use of the mentioned plant is safe up to 2000 mg/Kg BW as LD _50_ > 22,000 mg/Kg BW. The AuNPs expressed higher antioxidant (DPPH and ABTS), antimicrobial and antidiabetic activities than methanol extract. Eventually, it was concluded that the methanol extract of *Carduus edelbergii* Rech.f. and its fabricated AuNPs are valuable sources of antimicrobial, antioxidant and anti-diabetic compounds. Additional study is required to sharpen its pharmacological activities.

## Figures and Tables

**Table 1:** Chemical constituents found in the methanol extract of *Carduus edelbergii*.

Sr. No.	Name of Constituents	R. Time	Area	Concentration %	Kovait Index
1.	3,5-Dihydroxy-6-methyl-2,3-dihudro-4H-pyran-4-on	6.958	435,318	1.55	997
2.	Octanoic Acid	7.425	87,890	0.31	1012
3.	Octanoic Acid, ethyl ester	9.087	295,840	1.05	1060
4.	Hydroxymethylfurfurale	8.757	431,553	1.54	1051
5.	2-Decenal (E)-	10.107	651	0.00	1085
6.	Decanoic acid, ethyl ester	12.914	483,607	1.72	1157
7.	Tridecane	13.046	94,238	0.34	1160
8.	Hexadecanoic acid, ethyl ester	17.663	1,134,708	0.40	1260
9.	4-((1E)-3-Hydroxy-1-propenyl)-2-methoxyphenol	20.828	137,401	0.49	1328
10.	Tetradecanoic acid	21.316	136,039	0.48	1339
11.	Spiro[bicycle [3.3.0]octan-6one-3-cyclopropane]	21.720	125,990	0.45	1348
12.	9-Hexadecenoic acid, 1,4-methyl ester, (Z)-	24.331	117,568	0.42	1404
13.	Pentadecanoic acid, 1,4-methyl-, methyl ester	24.758	2,729,595	9.71	1414
14.	n-Hexadecanoic acid	25.552	4,235,069	15.07	1433
15.	13-Docosenoic acid, methyl ester, (Z)-	27.237	10,724,346	38.16	1472
16.	9,12-Octadecadienoic acid, methyl ester, (E, E)-	27.986	963,815	3.43	1488
17.	9-Octadecenoic acid, methyl ester, (E)-	28.125	4,422,512	15.72	1491
18.	11-Octadecenoic acid, methyl ester	28.20.8	360,740	1.28	1493
19.	Oleic Acid	28.913	2,208,215	7.86	1509

**Table 2:** Elemental composition of various elements in AuNPs sample.

Element	Weight%	Atomic%
C K	15.66	62.26
N K	2.07	7.04
O K	3.92	11.69
Au M	78.36	19.00
Total	100.00	

**Table 3:** Antibacterial zone of inhibition (mm) of extract and its mediated AuNPs.

Bacterial Species	Amoxicillin	AuNPs	Extract
*Klebsiella pneumoneae*	15.4 ± 0.2	12.3 ± 0.3	9.1 ± 0.7
*Staphylococcus epidermis*	16.2 ± 0.5	9.3 ± 0.4	8.4 ± 0.5
*Staphylococcus aureus*	14.1 ± 0.7	12.5 ± 0.6	8.5 ± 0.2
*Acetobacter spp*	15.3 ± 0.4	11.2 ± 0.6	8.7 ± 0.3
*Escherichia coli*	15.2 ± 0.3	10.7 ± 0.6	9.9 ± 0.5

**Table 4:** Antifungal potential (zone of inhibition: mm) of extract and its mediated AuNPs.

Bacterial Species	Terbinafine	AuNPs	Extract
*Aspergillus niger*	98.1 ± 1.9	92.4 ± 4.3	58.3 ± 2.7
*Aspergillus flavus*	97.5 ± 2.5	70.8 ± 5.5	62.28 ± 2.7

**Table 5:** Minimum inhibitory concentration (µg/mL) of extract and its mediated AuNPs.

Bacterial Species	Amoxicillin	AuNPs	Extract
*Klebsiella pneumoneae*	20.27	25.13	49.31
*Staphylococcus epidermis*	14.80	17.43	35.24
*Staphylococcus aureus*	1.35	2.50	11.25
*Acetobacter spp*	2.60	3.75	12.50
*Escherichia coli*	3.87	4.73	13.75

**Table 6:** Minimum inhibitory concentration of extract and its mediated AuNPs.

Bacterial Species	Terbinafine	AuNPs	Plant Extract
*Aspergillus niger*	6.31	9.75	13.50
*Aspergillus flavus*	3.23	5.74	10.75

**Table 7:** Percent antidiabetic potency of extract, glibenclamide and AuNPs.

S. No	Extract	Glibenclamide	AuNPs
250	50.4 ± 2.6	55.3 ± 4.2	50.2 ± 4.5
500	52.2 ± 4.2	58.5 ± 3.8	55.4 ± 2.7
750	57.7 ± 3.5	62.2 ± 4.3	59.3 ± 3.8
1000	60.8 ± 3.8	65.1 ± 3.2	63.7 ± 5.1

**Table 8:** Consequences on body weight of diabetic rats of a methanol extract and AuNPs.

Groups	Body Weight (g)			
	1st Day	7th Day	14th Day	21st Day
Normal	260.6 ± 5.71	262.1 ± 5.11	265.3 ± 4.21	267.1 ± 4.78
Diabetic control	265.0 ± 5.31	256.3 ± 5.21	246.0 ± 3.31	234.0 ± 3.56
Glibenclamide (10 mg/Kg BW)	264.5 ± 6.71	258.3 ± 5.32	259.2 ± 5.12 *	262.5 ± 4.70 *
Extract (150 mg/Kg BW)	263.3 ± 3.13	254.3 ± 3.22	255.2 ± 3.20	257.4 ± 6.05
Extract (300 mg/Kg BW)	268.5 ± 6.67	259.3 ± 7.04	262.0 ± 7.06 *	266.3 ± 6.54 *
AuNPs (5 mg/Kg BW)	260.1 ± 8.23	260.5 ± 6.89	261.1 ± 8.12 *	262.3 ± 8.20 *
AuNPs (10 mg/Kg BW)	275.5 ± 3.65	273.0 ± 3.68 *	276.7 ± 3.85 **	278.1 ± 4.22 **

The data (*n* = 5) are articulated as Mean ± SD, a significance is indicated at *p* < 0.05 (Dunnett’s-test); Comparison of the normal group was made with untreated diabetic group, and in turn, its relationship was established with groups treated with extract, AuNPs and standard. The level of significance is shown as * *p* < 0.05, ** *p* < 0.01.

**Table 9:** Effect of methanol extract and AuNPs on blood glucose.

Groups	Blood Glucose Level (mg/dL)			
	1st Day	7th Day	14th Day	21st Day
Normal	99.6 ± 3.50	100.4 ± 2.88	101.8 ± 3.27	102.8 ± 2.86
Diabetic control	272.8 ± 2.58	280.4 ± 2.30	290.2 ± 1.92	305.8 ± 3.03
Glibenclamide (10 mg/Kg BW)	272.8 ± 9.78	254.4 ± 9.07 *	224.2 ± 7.15 **	145.6 ± 3.20 **
Extract (150 mg/Kg BW)	290.6 ± 9.50	265.2 ± 7.69	182.4 ± 8.17 *	157.2 ± 3.11 *
Extract (300 mg/Kg BW)	286.0 ± 9.84	237.2 ± 8.07 **	177.4 ± 2.88 *	127.8 ± 8.19 **
AuNPs (5 mg/Kg BW)	282.4 ± 6.76	232.4 ± 7.02 **	164.6 ± 6.80 **	111.2 ± 6.72 ***
AuNPs (10 mg/Kg BW)	283.6 ± 8.20	229.6 ± 7.26 **	157.6 ± 5.50 **	102.0 ± 1.58 ***

The data (*n* = 5) are articulated as Mean ± SD, a significance is indicated at *p* < 0.05 (Dunnett’s-test); Comparison of the normal group was made with untreated diabetic group, and in turn, its relationship was established with groups treated with extract, AuNPs and standard. The level of significance is shown as * *p* < 0.05, ** *p* < 0.01 and *** *p* < 0.001.

**Table 10:** Effect of methanol extract and AuNPs on rat’s liver function.

Groups	ALT (µmol/L)	AST (µmol/L)	Total Bilirubin (µmol/L)	ALP (µmol/L)
Normal	140 ± 2.3	60 ± 2.23	1.04 ± 0.23	212 ± 3.68
Diabetic control	231 ± 2.9	102 ± 3.89	1.95 ± 0.34	411 ± 4.20
Glibenclamide (10 mg/Kg BW)	153 ± 3.6 **	91.3 ± 3.14	1.37 ± 0.05	293 ± 4.71 *
Extract (150 mg/Kg BW)	152 ± 2.8 **	84.7 ± 3.92 *	1.39 ± 0.10	299 ± 4.20 *
Extract (300 mg/Kg BW)	144 ± 5.1 ***	78.7 ± 5.35 **	1.28 ± 0.15 *	214 ± 6.50 ***
AuNPs (5 mg/Kg BW)	148 ± 8.1 ***	81.5 ± 3.96 *	1.32 ± 0.19	232 ± 5.13 **
AuNPs (10 mg/Kg BW)	141 ± 5.1 ***	71.6 ± 3.06 **	1.05 ± 0.11 **	209 ± 5.18 ***

The data (*n* = 5) are articulated as Mean ± SD, a significance is indicated at *p* < 0.05 (Dunnett’s-test); Comparison of the normal group was made with untreated diabetic group, and in turn, its relationship was established with groups treated with extract, AuNPs and standard. The level of significance is shown as * *p* < 0.05, ** *p* < 0.01 and *** *p* < 0.001.

**Table 11:** Effect of methanol extract and AuNPs on HDL cholesterol, triglycerides, total cholesterol, LDL cholesterol and serum VLDL cholesterol.

Groups	TG (mg/dL)	Cholesterol (mg/dL)	LDL (mg/dL)	VLDL (mg/dL)	HDL (mg/dL)
Normal	97 ± 1.5	89 ± 1.6	28 ± 2.3	20 ± 0.39	46 ± 5.3
Diabetic control	172 ± 1.3	94 ± 9.9	46 ± 2.1	33 ± 1.9	25 ± 1.4
Glibenclamide (10 mg/Kg BW)	137 ± 4.3 *	92 ± 3.1 *	33 ± 3.1 **	24 ± 0.6 *	35 ± 2.3 *
Extract (150 mg/Kg BW)	142 ± 4.5 *	93 ± 3.79 *	35 ± 4.1 *	25 ± 0.3	35 ± 3.9 *
Extract (300 mg/Kg BW)	121 ± 1.4 *	91 ± 5.1 **	32 ± 3.9 **	23 ± 0.32 *	42 ± 4.1 **
AuNPs (5 mg/Kg BW)	127 ± 4.17 *	92 ± 4.2 *	33 ± 3.2 **	24 ± 0.25 *	41 ± 4.5 **
AuNPs (10 mg/Kg BW)	99 ± 3.69 **	90 ± 3.1 **	29 ± 4.1 **	20. ± 0.83 **	45 ± 2.3 ***

The data (*n* = 5) are articulated as Mean ± SD, a significance is indicated at *p* < 0.05 (Dunnett’s-test); Comparison of the normal group was made with untreated diabetic group, and in turn, its relationship was established with groups treated with extract, AuNPs and standard. The level of significance is shown as * *p* < 0.05, ** *p* < 0.01 and *** *p* < 0.001.

**Table 12:** Effect of a methanol extract and AuNPs on creatinine and urea in rat’s serum.

Groups	Creatinine (mg/dL)	Urea (mg/dL)
Normal	0.66 ± 0.07	37.5 ± 1.40
Diabetic control	1.69 ± 0.27	52.0 ± 3.810
Glibenclamide (10 mg/Kg BW)	1.06 ± 0.27 *	41.3 ± 2.30 *
Extract (150 mg/Kg BW)	0.92 ± 0.72 *	43.8 ± 0.13 *
Extract (300 mg/Kg BW)	0.78 ± 0.04 **	39.4 ± 2.70 **
AuNPs (5 mg/Kg BW)	0.79 ± 0.20 **	38.8 ± 2.70 **
AuNPs (10 mg/Kg BW)	0.72 ± 0.10 ***	37.2 ± 3.06 **

The data (*n* = 5) are articulated as Mean ± SD, a significance is indicated at *p* < 0.05 (Dunnett’s-test); Comparison of the normal group was made with untreated diabetic group, and in turn, its relationship was established with groups treated extract, AuNPs and standard. The level of significance is shown as * *p* < 0.05, ** *p* < 0.01 and *** *p* < 0.001.

## Data Availability

Authors declare that all the data supporting the findings of this study are included in the article.

## Sample Availability

Samples of the compounds are not available from the authors.

## Abbreviations

UV-Vis	Ultraviolet-Visible
SEM	Scanning Electron Microscopy
EDX	Energy-dispersive X-ray spectroscopy
LDLc	Low-density lipoprotein
ALB	Albumin protein
ALP	Alkaline phosphatase
ALT	Alanine aminotransferase
C	Carbon
Ca	Calcium
Au	Gold
AuNPs	Gold nanoparticles
Cl	Chlorine
Create	Creatinine
ddH_2_O	Double-distilled water
FTIR	Fourier Transform Infrared spectroscopy
HDLc	High-density lipoprotein
NaOH	Sodium hydroxide
NIT	Nitrite
pH	Power of hydrogen ion
XRD	X-ray diffraction
BW	Body weight
